# A Rare Case of Extranodal Natural Killer/T-cell Lymphoma, Nasal Type Associated With Hemophagocytic Lymphohistiocytosis in a Patient With Recurrent Sinusitis

**DOI:** 10.7759/cureus.56237

**Published:** 2024-03-15

**Authors:** Bryce R Christensen, Chung-ting J Kou, Lauren E Lee

**Affiliations:** 1 Pulmonary and Critical Care Medicine, Mike O'Callaghan Military Medical Center, Nellis Air Force Base, USA; 2 Pulmonary and Critical Care Medicine, University of Nevada, Las Vegas, Las Vegas, USA; 3 Internal Medicine, Brooke Army Medical Center, Fort Sam Houston, USA; 4 Hematology and Oncology, Brooke Army Medical Center, Fort Sam Houston, USA

**Keywords:** hematology-oncology, active-duty military personnel, fevers, sinusitis, epstein-barr virus, extranodal natural killer/t-cell lymphoma, hemophagocytic lymphohistiocytosis

## Abstract

We present a rare case of hemophagocytic lymphohistiocytosis (HLH) secondary to nasal-type extranodal natural killer/T-cell lymphoma (ENKL). Nasal-type ENKL is a rare subtype of non-Hodgkin's lymphoma usually associated with Epstein-Barr virus (EBV).

The patient was a 19-year-old woman who presented with facial numbness, diminished hearing, and dysgeusia. She was febrile with palatal necrosis, loss of gag reflex, and cranial nerve palsies. Labs revealed neutropenia. Broad-spectrum antimicrobials, including amphotericin, were started. Given concern for invasive fungal disease, she underwent surgical debridement, which revealed inflamed fibrous tissue and extensive necrosis. Pathology showed no fungal elements or malignancy. Lack of clinical improvement and worsening palatal necrosis prompted additional debridement. Histology identified an atypical CD3+/CD56+ cellular infiltrate. Bone marrow biopsy showed prominent hemophagocytosis, but no malignancy. She met the criteria for HLH and high-dose dexamethasone was started. Her fevers resolved. Additional labs and nasal tissue sampling with EBV-encoded RNA staining were recommended. Flow cytometry was negative, but histology revealed ENKL nasal-type, with positive EBV-encoded RNA in situ hybridization. Plasma EBV DNA level was 11,518 IU/mL. The M-SMILE (dexamethasone, methotrexate, ifosfamide, l-asparaginase, and etoposide) regimen was initiated; one cycle led to marked improvement. EBV level returned to zero. Subsequent radiation and chemotherapy, followed by autologous stem cell transplant consolidation, led to complete remission.

We conclude that ENKL may mimic invasive sinusitis clinically. Fibrinoid necrosis in vessels and surrounding tissues often leads to diagnostic delay. It is important to have a high degree of clinical suspicion for malignancy in cases of HLH and sinusitis unresponsive to appropriate therapy. Obtaining proper tissue, communication with the pathologist, and prompt initiation of therapy are crucial.

## Introduction

Hemophagocytic lymphohistiocytosis (HLH) is a rare, life-threatening syndrome caused by pathologic activation of the immune system [1]. While most commonly associated with inherited HLH gene mutations in pediatric patients [2], it may also be sporadic and associated with autoimmune disease, infection, or malignancy [3]. Genetic defects for familial HLH frequently map to genes coding for cytotoxic granule formation [4,5]. *PRF1 *- a gene encoding perforin - was one of the first genes discovered to be associated with familial HLH; other associated mutations include *UNC13D*, *STXBP2*, *Rab27a*, *STX11*, *SH2D1A*, and *XIAP *[3].

The pathogenesis of HLH involves macrophages, natural killer (NK) cells, and cytotoxic lymphocytes [1]. The disease results from a failure of regulatory pathways that terminate inflammatory responses. The immune system removes cells that are either unnecessary or dangerous. However, the immune system can be tempered to avoid the removal of useful cells [6]. When downregulation of the immune system fails, the resulting hyperinflammatory state can lead to HLH [6]. Symptoms may include splenomegaly, hepatomegaly, and fevers. Cytopenias, elevated triglycerides, and decreased fibrinogen may also be apparent [3]. Hemophagocytosis may be present on bone marrow biopsy, but this is not pathognomonic and may be present in as few as 25% of cases [7].

In a study of 369 patients with HLH, Bergsten et al. [8] showed the presence of fever (95% of patients), splenomegaly (89%), bicytopenia (92%), hypertriglyceridemia or hypofibrinogenemia (90%), hemophagocytosis (82%), ferritin > 500 mcg/L (94%), low to absent NK cell activity (71%), and elevated soluble CD25 elevation (97%). Patients with HLH may present with a sepsis-like picture with fever and performance status deterioration.

The results of the HLH-2004 trial led to the eight diagnostic criteria for HLH: temperature ≥ 38.3°C, splenomegaly, cytopenias (hemoglobin < 9 g/dL for adults, platelets less than 100 x 103/mL, or neutrophils less than 1 x 103/mL) affecting at least two of three lineages in peripheral blood, either hypertriglyceridemia (>265 mg/dL) or hypofibrinogenemia (<150 mg/dL), hemophagocytosis found in the bone marrow, spleen, lymph nodes, or liver, low to absent NK cell activity (<10 lytic units), ferritin > 500 ng/mL, and soluble CD25 ≥2400 U/mL [3,8-10]. Genetic testing may demonstrate homozygous HLH-associated mutations in children or heterozygous HLH-associated mutations with clinical findings of HLH in adults [2,11] in cases of primary HLH. Additionally, CXC chemokine ligand (CXCL) levels have been utilized as novel markers to help distinguish lymphoma-associated HLH from sepsis, as well as markers of response to treatment [12,13].

Unfortunately, some patients with HLH do not meet the above criteria, and untreated HLH carries significant mortality. As such, the diagnosis can sometimes be presumed - and treatment initiated - if clinical suspicion for HLH is high [1]. The Histiocyte Society provided modified HLH diagnostic criteria in 2009, which require any of the following three criteria: molecular diagnosis; three out of the four findings: fever, splenomegaly, cytopenias with a minimum of two cell lines reduced, and/or hepatitis; and presence of at least one of the following: hemophagocytosis, elevated ferritin, elevated CD25, and/or absent or very decreased NK function. The other findings of hypertriglyceridemia, hypofibrinogenemia, and hyponatremia are supportive of HLH diagnosis, but not required [1].

Treatment of HLH dramatically increases survival from only months without treatment, to five or more years with treatment [8,10,14]. Therapeutic regimens often include dexamethasone and etoposide, in addition to allogeneic hematopoietic stem cell transplantation (HSCT) in patients with genetic, relapsing, severe, or persistent disease [8,10,14]. Intrathecal methotrexate and hydrocortisone are also used in patients with involvement of the central nervous system [8,10,14].

The patient presented in this report is one such case where HLH diagnosis was difficult. The suspected culprit initially for her presentation was infection resulting in a delayed diagnosis. Initiation of treatment led to a dramatic improvement in her disease-related symptoms. We present this case to highlight the diagnostic and therapeutic challenges of HLH.

## Case presentation

A 19-year-old woman presented to the emergency room with acute-on-chronic sinusitis, nasal obstruction, and left periorbital swelling with purulent discharge. Computed tomography (CT) of the sinuses showed pansinusitis with findings suggestive of preseptal cellulitis. She was admitted for empiric board-spectrum antimicrobials and functional endoscopic sinus surgery with bilateral middle meatal antrostomies.

Due to persistent and progressive symptoms despite completion of appropriate antimicrobial treatment, a repeat CT of the sinus and face was obtained. CT of the sinus and face demonstrated a medial canthus abscess with left periorbital cellulitis and intraorbital myositis. The patient developed left-sided facial and palatal numbness, diminished left-sided hearing, and loss of gag reflex. The patient was transferred to a large military treatment facility for further management. Upon arrival, her vitals were normal. The exam showed significant left eye edema impairing her ability to open her eye independently, impaired sensation to light touch in the left maxillary branch of the trigeminal nerve distribution, diminished left-sided hearing, and a palate that did not elevate. Laboratory assessment (Table 1) revealed a white blood cell (WBC) count of 1,930 per microliter (μL), absolute neutrophil count (ANC) of 1,310 per μL, and thrombocytopenia with platelets of 114,000 per μL. Liver enzymes were elevated, with aspartate aminotransferase (AST) of 101 units per liter (U/L) and alanine aminotransferase (ALT) of 112 U/L. Ferritin was elevated at 1,788 nanograms per milliliter (ng/mL).

**Table 1:** Patient's laboratory values

Laboratory study	Value	Reference range
White blood cell (WBC)	1,930 WBC/μL	4,500 to 11,000 WBCs/μL
Absolute neutrophil count (ANC)	1,310 ANC/μL	2,500 to 6,000 ANC/μL
Platelets	114,000 platelets/μL	150,000 to 450,000 platelets/μL
Aspartate aminotransferase (AST)	101 U/L	≤33 U/L
Alanine aminotransferase (ALT)	112 U/L	10 to 49 U/L
Ferritin	1,788 ng/mL	12 to 150 ng/mL
Epstein-Barr virus quantitative level	11,518 IU/mL	0 IU/mL

**Figure 1:**
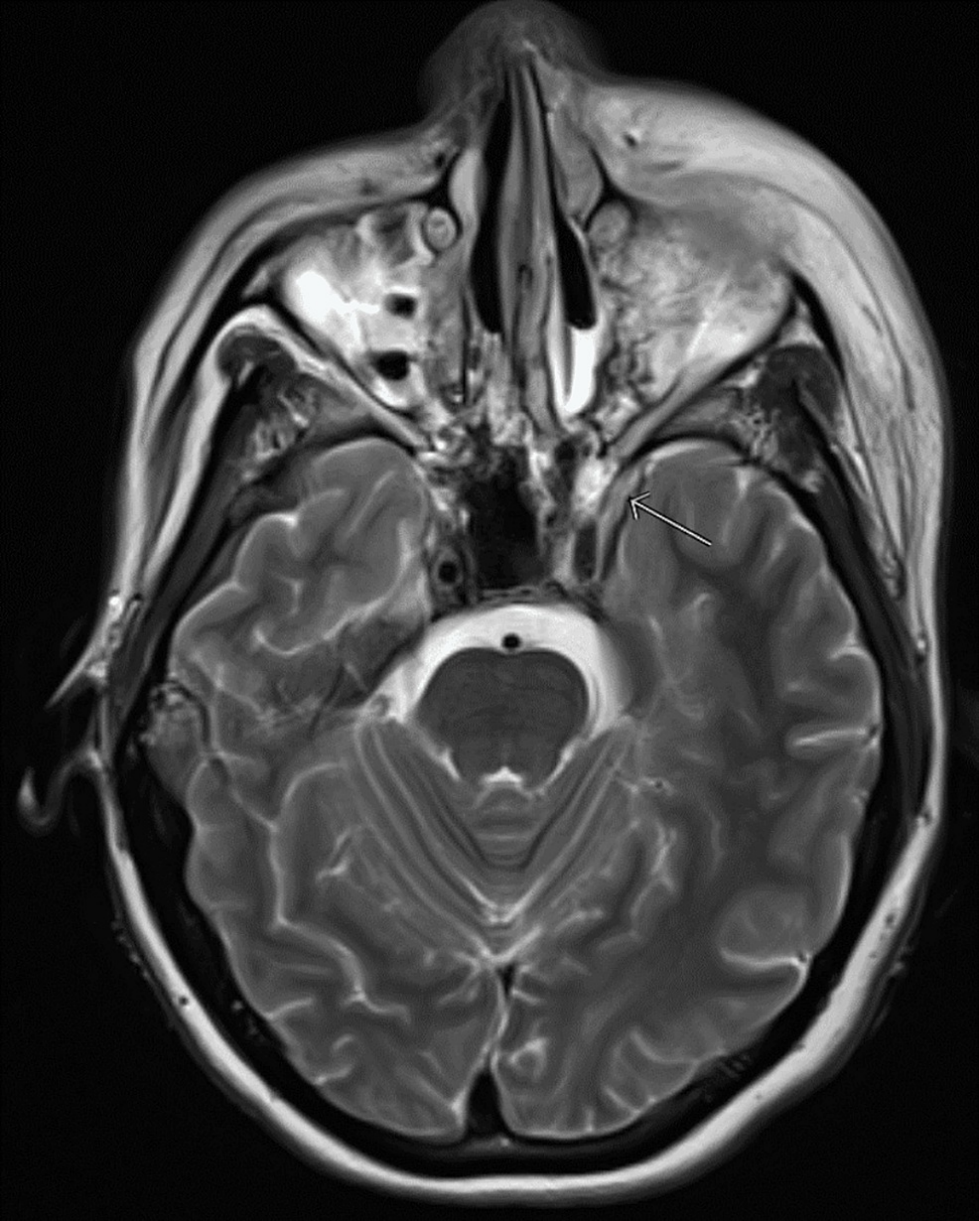
MRI depicting intracranial findings

Interval CT imaging of the face, chest, abdominal, and pelvis was repeated and was consistent with prior imaging concerning for invasive fungal sinusitis. Brain magnetic resonance imaging (MRI) revealed mucosal non-enhancement in the left maxillary and left sphenoid sinus cavities, with adjacent inflammatory findings suggestive of invasive fungal sinusitis with possible early epidural involvement (Figure 1). Magnetic resonance venography was normal. She underwent extensive debridement by otolaryngology. Broad-spectrum antibiotics as well as voriconazole and amphotericin were initiated for suspected invasive bacterial/fungal infections. A transthoracic echocardiogram demonstrated a mitral valve mobile density concerning for infective endocarditis secondary to a hematogenous infection.

During the patient’s hospitalization, she developed transfusion-dependent anemia and neutropenia without an identifiable etiology. Hematology/oncology was consulted and a bone marrow biopsy was performed. Neutropenia was initially supported with filgrastim with an adequate response. A maxillary sinus tissue culture from the initial debridement was negative for fungus. However, methicillin-sensitive *Staphylococcus aureus* and *Streptococcus sanguinis* were identified on the maxillary sinus tissue culture. The patient’s empiric antimicrobials were de-escalated accordingly based on tissue culture data. Despite these interventions, the patient’s status did not improve, prompting a repeat nasal endoscopy and debridement with the goal of improving access for nasal irrigation and obtaining additional diagnostic tissue. Interval CT of the chest, abdomen, and pelvis showed nonspecific hepatosplenomegaly without lymphadenopathy.

**Figure 2:**
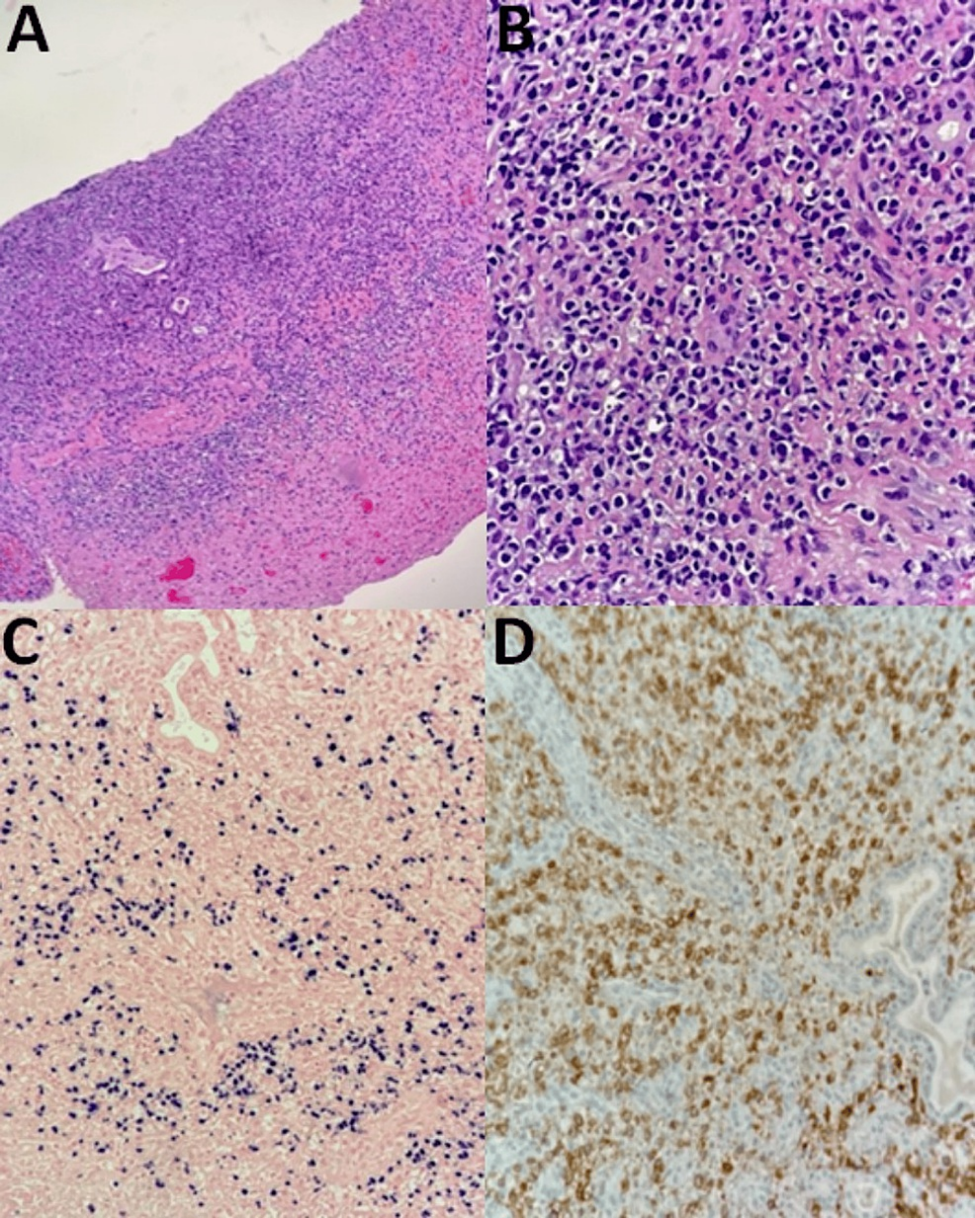
Histology slides (panel 1 of 2) A: Nasopharynx biopsy: hematoxylin and eosin stain, 10x magnification. B: Nasopharynx biopsy: hematoxylin and eosin stain, 40x magnification. C: Nasopharynx biopsy: Epstein-Barr virus positivity, 20x magnification. D: Nasopharynx biopsy: CD56+, 20x magnification.

**Figure 3:**
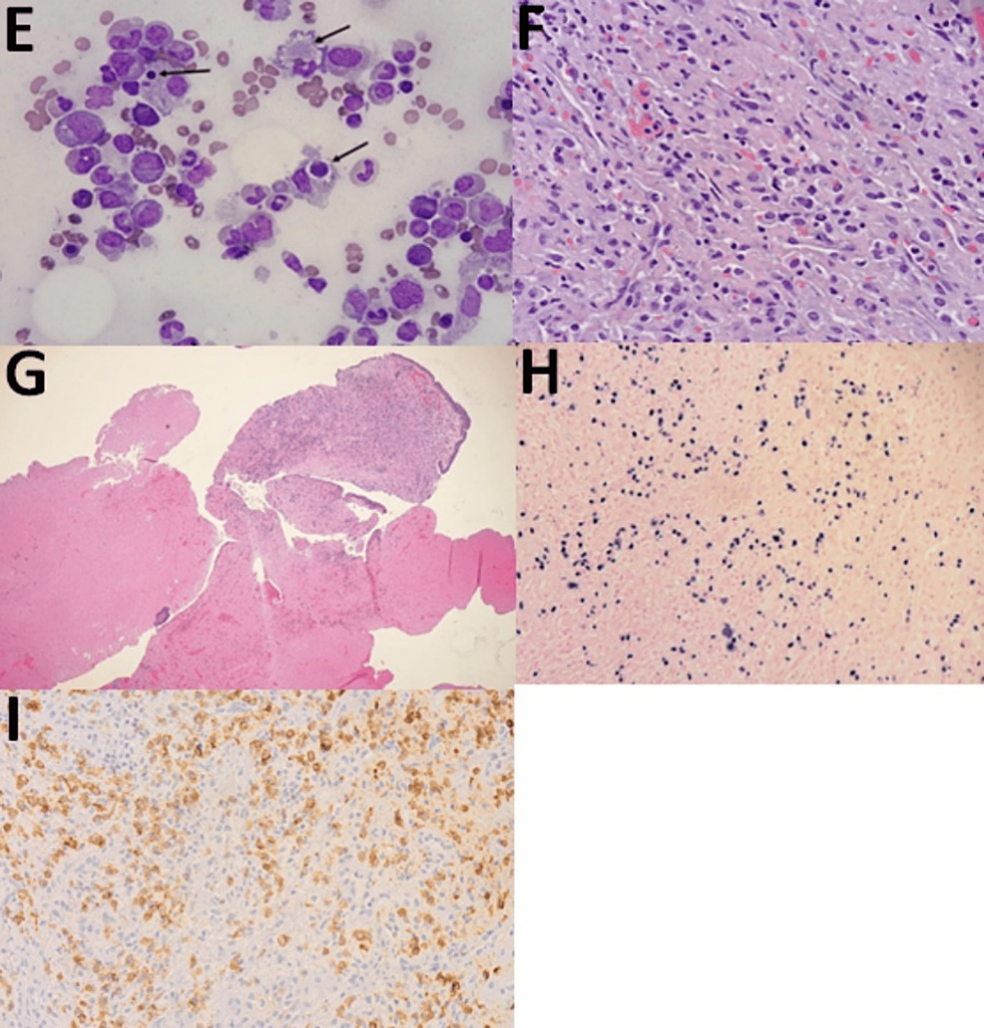
Histology slides (panel 2 of 2) E: Bone marrow biopsy: Wright-Giemsa, 1000x magnification. This bone marrow aspirate on oil shows prominent hemophagocytic cells, indicated by arrows. F: Nasopharynx biopsy: hematoxylin and eosin stain, 400x magnification. G: Nasopharynx biopsy: hematoxylin and eosin stain, 40x magnification. Nasal curetting showed bone and respiratory mucosa with prominent necrotic material. H: Nasopharynx biopsy: Epstein-Barr virus positivity, 200x magnification. This is an Epstein-Barr virus in situ hybridization. The neoplastic cells are positive for the Epstein-Barr virus. I: Nasopharynx biopsy: CD56+, 200x magnification.

The bone marrow biopsy demonstrated hemophagocytosis and serum interleukin-2 receptor (IL-2R) was elevated. In the clinical context of fevers, hepatosplenomegaly, cytopenias, elevated ferritin, elevated IL-2R, and hemophagocytosis on bone marrow biopsy, high-dose steroids were initiated for suspected HLH secondary to bacterial infection. Germline testing for primary HLH was obtained and negative for genetic HLH. A transesophageal echocardiogram was performed and was negative for vegetation. Although HLH was initially attributed to a bacterial sinus infection, additional operative debridement by otolaryngology was performed given HLH’s association with malignancy and other non-infectious etiologies. Flow cytometry of nasal tissue was non-diagnostic due to inadequate tissue sampling. Flow cytometry of cerebral spinal fluid was negative for malignancy. Immunohistochemistry of the right posterior ethmoid biopsy demonstrated CD56, CD3, and Epstein-Barr virus (EBV) positivity with myogenin and anaplastic lymphoma kinase (ALK) negativity, consistent with extranodal NK/T-cell lymphoma (ENKL), nasal-type (Figures 2, 3).

Staging positron emission tomography (PET)-CT demonstrated abnormal fluorodeoxyglucose (FDG) avidity of the left orbital, paranasal sinuses, nasopharynx, and enlarged level two lymph nodes. Her serum EBV quantitative level was 11,518 international units per milliliter (IU/mL) (Table 1), portending a poorer prognosis. Human T-cell lymphotropic virus (HTLV) was negative, and cerebrospinal fluid assessment results were negative. The patient was staged as advanced disease given skull involvement complicated by neurological deficits and HLH. An anti-neoplastic regimen with dexamethasone, methotrexate, ifosfamide, l-asparaginase, and etoposide (modified-SMILE regimen) was started. Five cycles of modified-SMILE were administered with a total of 5000 centigray over 20 fractions of radiation therapy to the maxillary sinus region. The patient achieved a complete remission based on an undetectable serum EBV, negative nasal biopsy, and resolution of cranial nerve deficits. Surveillance MRI demonstrated a continued response to therapy with a small amount of left anterior extraconal orbital disease, with left interior and medial rectus muscle thickening suspected secondary to inflammation versus scarring. The patient proceeded with high-dose BEAM (carmustine, etoposide, cytarabine, and melphalan) conditioning followed by autologous bone marrow rescue. The patient continued to be in remission over two years after autologous bone marrow transplant.

## Discussion

This patient presented with an uncommon disease that can mimic other pathologic processes, making diagnosis challenging. She presented with recurrent sinusitis that failed to improve with debridement and antimicrobial therapy, developed HLH, and was ultimately found to have ENKL, nasal-type.

HLH is a rare entity, with one study estimating an annual incidence of only one in 800,000 persons per year [15]. As previously mentioned, HLH can be primary, but approximately 90% of the time is secondary to infection or malignancy [15,16]. In fact, approximately 1/3 of patients test positive for EBV [16]. Further complicating this case was the fact that the patient fell into an age range where primary HLH was a consideration. Germline testing was negative but should be considered in cases where there is diagnostic uncertainty.

The morbidity and mortality of untreated HLH is high, with long-term survival rates as low as 4% without treatment [14]. Treatment can drastically improve survival, with three to five-year survival rates after treatment greater than 50% [14,16]. The diagnosis of HLH can be difficult, and as demonstrated with our patient, is frequently mistaken for infection [17].

ENKL, nasal-type, is an aggressive T-cell lymphoma, which accounts for 10% of all peripheral T-cell lymphomas in North America. ENKL has an increased incidence among Asian countries [18]. The median survival is 31 months for limited disease and six months for advanced disease based on data from the National Cancer Institute's Surveillance, Epidemiology, and End Results (SEER-18) between 2000 and 2018 [19].

Workup of ENKL includes dedicated imaging of nasal sinuses by either MRI or CT, as well as nasal endoscopy to assess for localized versus extra-nasal disease. Multiple biopsies may be necessary to establish a diagnosis, as extensive necrosis can obscure the results. The classic histological presentation of ENKL is extensive angioinvasion with necrosis and positive staining for CD2, CD56, cytoplasmic CD3, but not surface CD3, and cytotoxic markers [20]. Tissue biopsies must demonstrate the presence of EBV-encoded RNA by ISH [20].

Plasma EBV DNA should be obtained at the time of diagnosis and surveilled through treatment given its role in prognosis [20,21]. EBV DNA in the plasma has a higher specificity and sensitivity for EBV-positive disease as compared to EBV in peripheral blood mononuclear cells (PBMC), as demonstrated in a retrospective study of 2142 patients at Johns Hopkins Hospital clinical laboratory by Kanakry et al. [22]. EBV DNA is derived from the plasma in patients with EBV-positive tumors, as the malignant cells can circulate within the plasma, and EBV DNA levels obtained in a PBMC sample only reflect a fraction of circulating EBV tumor cells [22]. A prognostic model for ENKL, called the Prognostic Index for Natural Killer Lymphoma (PINK-E), risk stratifies patients into the following groups: low-risk (no risk factors), intermediate-risk (one risk factor), or high-risk (three or more risk factors) groups. The risk factors are: (1) age older than 60 years, (2) stage III or IV disease, (3) distant lymph node involvement, (4) non-nasal-type disease, and (5) detectable peripheral blood EBV titers [20,23]. The three-year overall survival rates associated with PINK-E low-risk, intermediate-risk, and high-risk categories are 81%, 55%, and 28%, respectively [23].

Due to extensive necrotic tissue mixed with hemorrhagic cells and inflamed respiratory mucosa on the initial nasal biopsy, our patient required two additional biopsies to establish extranodal NK/T-cell lymphoma, nasal-type, complicated by HLH. Our patient was considered low-risk based on the PINK-E model, with her only risk factor being a detectable plasma EBV DNA of 11,518 copies/mL. However, we elected to treat the patient as advanced disease, given her presentation with cranial nerve deficits secondary to mass effect and concomitant HLH.

Treatment of advanced disease consists of chemotherapy, but consolidation with radiation therapy (RT) or autologous HSCT may provide additional benefits [20,24,25]. NK cells express high concentrations of multidrug-resistant P-glycoprotein, making anthracycline-containing regimens ineffective [26]. Chemotherapy options include SMILE (dexamethasone, methotrexate, ifosfamide, l-asparaginase, and etoposide), which is administered every 28 days. Less toxic alternatives such as DDGP (dexamethasone, cisplatin, gemcitabine, and pegaspargase), AspaMETDex, and GemOX have also been studied [20,27]. SMILE treatment was initially evaluated in advanced and relapsed disease, with a high rate of objective response rate (ORR) of 79% in a phase II trial by Yamaguchi et al. of 38 eligible patients after two cycles [28]. However, there were high toxicity rates, with grade four neutropenia being the most common at 92%. Other grade four toxicities include infection (n = 6), hyperbilirubinemia (n = 1), alanine transaminase elevation (n = 2), and encephalopathy (n = 1) [28]. Due to the high risk of morbidity and mortality, SMILE is avoided in patients with poor performance status, lymphopenia (<500/uL lymphocytes), or a high tumor burden [20].

In limited-stage ENKL, RT is associated with improved overall survival (OS), as defined by prospective trials [29,30]. However, the benefit of radiation is less obvious in advanced diseases. Bi et al. demonstrated that post-chemotherapy radiotherapy was associated with a two-year OS of 57.5% vs. 14.5% compared to chemotherapy alone in patients with stage III or IV NK/T-cell lymphoma [24]. A modified SMILE (mSMILE) regimen consisting of dexamethasone, methotrexate, ifosfamide, peg-asparaginase, and etoposide given every three weeks followed by RT with 45 gray (Gy) has been adopted by Memorial Sloan Kettering Center [31]. A 21-day cycle was adopted to provide patients the opportunity to quickly transition to RT after two cycles of mSMILE [31]. Table 2 compares the SMILE and mSMILE regimens.

**Table 2:** SMILE vs. modified SMILE regimen Adapted from Ghione P, Qi S, Imber BS, et al. Modified SMILE (mSMILE) and intensity-modulated radiotherapy (IMRT) for extranodal NK-T lymphoma nasal type in a single-center population. Leuk Lymphoma. 2020;61(14):3331-3341. DOI: 10.1080/10428194.2020.1811864.

	Original SMILE	Modified SMILE
Medication 1	Methotrexate 2g/m^2^ (with leucovorin 15 mg) days 1, 2, 3, and 4	Methotrexate 2g/m^2^ (with leucovorin 25 mg) days 1, 2, 3, and 4
Medication 2	Ifosfamide 1,500 mg/m^2^ (with mesna 300 mg/m^2^) days 2, 3, and 4	Ifosfamide 1,500 mg/m^2^ (with mesna 300 mg/m^2^) days 2, 3, and 4
Medication 3	Dexamethasone 40 mg/d days 2, 3, and 4	Dexamethasone 40 mg/d days 2, 3, and 4
Medication 4	Etoposide 100 mg/m^2^ days 2, 3, and 4	Etoposide 100 mg/m^2^ days 2, 3, and 4
Medication 5	L-asparaginase 6000 U/m^2^ days 8, 10, 12, 14, 16, 18, and 20	Peg-asparaginase 1500 U/m^2^ ~ day 7 (range: from day 5 to 31)
Cycle duration	28 days	21 days
Radiotherapy	None	45 Gray
Expected cycles	6	2 (localized disease), 3-4 (advanced disease)

Ghione et al. demonstrated with mSMILE an ORR of 100% for stage III-IV ENKL, as well as an OS of 100% at the median follow-up at 31 months for both limited and extensive stage ENKL [31]. Our 19-year-old patient completed five cycles of mSMILE with grade two mucositis, oral candidiasis, and *Clostridioides difficile* infection. She received RT to the maxillary sinus region with 45 Gy over 20 fractions between cycles three and four of SMILE. After completion of chemotherapy and RT, the patient was in complete remission as indicated by an undetectable plasma EBV DNA, negative endoscopic biopsies, and imaging.

The role of HSCT is well established in lymphomas such as relapsed diffuse large B-cell lymphoma and relapsed classic Hodgkin's lymphoma. The role of HSCT in ENKL is less clear. A multinational, multicenter, matched controlled retrospective analysis of 59 patients with ENKL from Japan, Korea, and Hong Kong found a significant survival advantage with autologous HSCT in patients with advanced disease who achieve a clinical remission (CR) at the time of transplantation [32]. A similar retrospective study of ENKL patients in the Western hemisphere by Fox et al. demonstrated better two-year disease-free survival in patients who achieved CR at the time of autologous HSCT compared to patients not in CR [33]. Autologous HSCT is routinely not recommended in limited disease, as the Lee et al. matched analysis demonstrated no improvement in limited disease patients [32]. The American Society of Blood and Marrow Transplantation supports the use of autologous HSCT in relapsed chemo-sensitive localized NK/T-cells [34]. Autologous HSCT (strongly) and allogenic HSCT (weakly) are recommended for disseminated NK/T-cell as front-line consolidation and chemo-sensitive relapsed disseminated disease [34]. Allogenic HSCT is weakly recommended for primary relapsed or relapsed refractory disease [34]. Though our patient’s PET-CT imaging was consistent with contiguous stage IIE disease, she was treated as an advanced disease patient with concurrent cranial nerve deficits and HLH at presentation. The patient underwent BEAM conditioning with autologous HSCT rescue after achieving CR with mSMILE and RT. Two years after mSMILE with RT followed by consolidative autologous HSCT, the patient remained in CR.

## Conclusions

We report a case of a patient presenting with recurrent sinusitis who was ultimately diagnosed with ENKL after multiple tissue biopsies with a hematological course complicated by concomitant HLH. Early recognition of HLH and treatment of the underlying etiology is imperative to reducing its high morbidity and mortality. Our case highlights ENKL, a rare subtype of lymphoma caused by EBV, as an etiology of HLH. Pathologic diagnosis of ENKL is challenging due to extensive necrosis, which often necessitates multiple biopsies to establish a diagnosis. Limited-stage ENKL is managed with asparaginase-based chemotherapy with radiation therapy. The optimal treatment for advanced and relapsed ENKL is less defined. Further studies are needed to elucidate the role of hematopoietic stem cell transplantation for advanced and relapsed ENKL.
